# Novel sequence type 2040 non-O1/non-O139 *Vibrio cholerae* from southern China: genomic and phenotypic features with molecular epidemiological implications

**DOI:** 10.3389/fmicb.2026.1955993

**Published:** 2026-10-15

**Authors:** Yuanzhi Xia, Jiawei Ding, Jinyan Kai, Chunhong Xie, Yanqin Nie, Jiaqiu Ru, Lianjiang Huang, Juan Wen

**Affiliations:** 1 Second Affiliated Hospital of Xiamen Medical College, Xiamen, China; 2 Yan’an Hospital Affiliated To Kunming Medical University, Kunming, China; 3 Xiamen Center for Disease Control and Prevention, Xiamen, China

**Keywords:** antimicrobial susceptibility, comparative genomics, non-O1/non-O139 *Vibrio cholerae*, sequence type 2040 (ST2040), virulence factors, whole-genome sequencing

## Abstract

**Introduction:**

Non-toxigenic non-O1/non-O139 *Vibrio cholerae* (NOVC) are opportunistic aquatic pathogens that cause extraintestinal infections including bacteremia, particularly in patients with underlying comorbidities. The incidence of NOVC bacteremia has increased in coastal China, yet the genomic diversity and population structure of clinical NOVC isolates remain poorly defined. This study aimed to characterize a clinical NOVC isolate from southern China at both phenotypic and genomic levels.

**Methods:**

We recovered a clinical NOVC isolate (HL2601) from a bloodstream infection case in Xiamen, China. Antimicrobial susceptibility was determined by broth microdilution and disk diffusion. Whole-genome sequencing was performed, followed by multilocus sequence typing (MLST), average nucleotide identity (ANI) analysis, core-genome phylogeny reconstruction, virulence and resistance gene profiling, and quantitative genome collinearity and structural variation analysis.

**Results:**

The isolate was confirmed as *V. cholerae* and assigned to a novel sequence type, ST2040. It lacked cholera toxin and toxin-coregulated pilus genes but harbored multiple alternative virulence determinants, including intact type II and type VI secretion systems, RTX toxin, hemolysins, and iron uptake systems. HL2601 was susceptible to all 11 tested antibiotics and clustered phylogenetically with extraintestinal clinical and environmental NOVC strains, suggesting a potential environmental reservoir origin.

**Discussion:**

These findings expand the known genomic diversity of clinical NOVC in China. They provide a genomic baseline for surveillance of emerging NOVC clones and inform empirical antibiotic selection for extraintestinal vibriosis in coastal regions.

## Introduction

*Vibrio cholerae* causes cholera, a disease that remains a persistent global health challenge. Toxigenic O1 and O139 serogroups drive pandemic transmission and contribute substantially to morbidity and mortality (Clemens et al., 2017). The World Health Organization estimates 1.3–4.0 million cholera cases and 21,000–143,000 deaths each year (World Health Organization, 2025a). Since 2021, a marked global resurgence of cholera has been documented. Geographic coverage has expanded, and case-fatality rates have risen sharply (World Health Organization, 2025b). This re-emergence underscores the continuing threat of this ancient disease.

Non-toxigenic non-O1/non-O139 *V. cholerae* (NOVC) differ fundamentally from their toxigenic counterparts. They are ubiquitous in aquatic environments and have emerged as opportunistic pathogens (Zhang et al., 2020). Their clinical spectrum includes bacteremia, wound infections, and peritonitis (Deshayes et al., 2015). Unlike O1/O139 strains, NOVC typically lack the cholera toxin gene (ctxAB) and the toxin-coregulated pilus (tcp). Yet they may carry alternative virulence factors that confer pathogenic potential in immunocompromised hosts (Gherlan et al., 2025; Zhang et al., 2024). Climate change, warming coastal waters, and rising seafood consumption have been linked to increasing NOVC infections (Vezzulli et al., 2020). This trend poses a particular concern for coastal regions worldwide.

In China, NOVC bacteremia has drawn increasing attention over the past two decades. Li et al. (2020) identified 82 cases between 2005 and 2019. Most patients had underlying conditions–liver cirrhosis, diabetes, or immunocompromised states. Mortality was substantial in this cohort. These findings highlight the clinical importance of NOVC in vulnerable populations. However, the population structure, genomic diversity, and evolutionary dynamics of clinical NOVC isolates in China remain largely underexplored.

In this study, we performed comprehensive genomic and phenotypic characterization of a clinical NOVC isolate (HL2601) recovered from a bacteremia patient in Xiamen, a coastal city in southern China. We conducted whole-genome sequencing, multilocus sequence typing (MLST), average nucleotide identity (ANI) analysis, core-genome phylogenetic reconstruction, virulence and resistance gene profiling, and quantitative genome collinearity and structural variation analysis. Our findings expand the current understanding of NOVC genetic diversity in China and provide a basis for strengthening regional surveillance and optimizing clinical management of extraintestinal vibriosis.

## Materials and methods

### Bacterial isolation and identification

The *V. cholerae* isolate HL2601 was recovered from a blood culture of a 56-years-old male patient with biliary tract infection-associated bacteremia admitted to a tertiary hospital in Xiamen, China. Blood specimens were inoculated into BacT/ALERT FA Plus aerobic culture bottles and incubated in the BacT/ALERT 3D automated microbial detection system (bioMérieux, Marcy-l’Étoile, France) at 35 °C with continuous agitation. Upon positive growth signaling, the broth was subcultured onto Columbia blood agar and MacConkey agar plates (Autobio Diagnostics, Zhengzhou, China) and incubated aerobically at 35 °C for 18–24 h. Colonies with typical *Vibrio* morphology were selected for isolate purification. Preliminary species identification was performed by matrix-assisted laser desorption/ionization time-of-flight mass spectrometry (MALDI-TOF MS, Microflex LT, Bruker Daltonik, Bremen, Germany) with a species-level confidence threshold of ≥2.0. O1 and O139 serogrouping was performed using the standard slide agglutination method with commercial diagnostic antisera (Denka Seiken Co., Ltd., Tokyo, Japan). This study was approved by the institutional ethics committee via expedited review, with a waiver of informed consent.

### Antimicrobial susceptibility testing

Antimicrobial susceptibility testing was performed following the Clinical and Laboratory Standards Institute (CLSI) guideline M45 for infrequently isolated or fastidious bacteria (Clinical and Laboratory Standards Institute, 2015). The following 11 antimicrobial agents were tested: amikacin, ampicillin, ampicillin-sulbactam, cefepime, ceftazidime, ciprofloxacin, gentamicin, imipenem, levofloxacin, piperacillin-tazobactam, and trimethoprim-sulfamethoxazole.

All agents were tested by broth microdilution except imipenem (Kirby-Bauer disk diffusion). *Escherichia coli* ATCC 25922 and *Pseudomonas aeruginosa* ATCC 27853 were included as quality control strains in each test run. MIC values were interpreted according to CLSI M100 clinical breakpoints (Clinical and Laboratory Standards Institute, 2022).

### Genomic DNA extraction, whole-genome sequencing and genome annotation

The *V. cholerae* isolate HL2601 was streaked onto blood agar plates and incubated at 35 °C for 18 h. A single purified colony was inoculated into LB broth and cultured overnight at 35 °C with constant shaking. Genomic DNA was extracted using the TIANamp Bacteria DNA Kit (Tiangen Biotech, Beijing, China) following the manufacturer’s instructions.

Whole-genome sequencing was performed on the Illumina NovaSeq 6000 platform (Illumina, San Diego, USA) using a 2 × 150 bp paired-end sequencing protocol. Raw reads were quality-filtered using fastp v0.19.6 (Chen et al., 2018), and *de novo* genome assembly of clean reads was performed using SPAdes v3.15.4 (Bankevich et al., 2012). The raw sequencing reads generated in this study have been deposited in the NCBI Sequence Read Archive (SRA) under BioProject accession PRJNA1479141, BioSample accession SAMN60942877, and SRA run accession SRR39195233.

Protein-coding sequences were predicted using Prokka v1.14.6 (Seemann, 2014). Transfer RNA (tRNA) and ribosomal RNA (rRNA) genes were annotated using tRNAscan-SE (Lowe and Eddy, 1997) and Barrnap v0.9^1^, respectively.

Putative virulence factors were identified by sequence alignment against the Virulence Factor Database (VFDB) (Liu et al., 2019), with screening thresholds set at ≥90% nucleotide identity and ≥80% query coverage. Detected genes were categorized into functional classes, and the completeness of each virulence system was evaluated based on the presence of all core structural and functional genes. To rule out key toxigenic determinants not fully covered by VFDB annotation, targeted BLASTn searches for ctxAB, tcpA, zot, chxA, and stn were performed with relaxed thresholds (≥70% identity, ≥50% query coverage). The full per-gene list of virulence factors, including functional description, nucleotide identity, query coverage and genomic location, is provided in Supplementary Table 1. Acquired antimicrobial resistance genes were detected using ResFinder 4.1^2^ (Bortolaia et al., 2020).

For comparative virulence profiling, five representative non-O1/non-O139 *V. cholerae* (NOVC) genomes were retrieved from the NCBI GenBank database for comparison, including one clinical blood isolates (strain 973360, accession no. GCF_032841475.3), one clinical bile isolate (strain C3616, accession no. GCF_008369605.1), one environmental water isolate (strain E1, accession no. GCF_026013235.1), one aquaculture water isolate (strain Vc473, accession no. GCF_041447385.1), and one aquaculture shrimp isolate (strain GXFL1-4, accession no. GCF_021431945.1). Presence/absence profiles of nine key virulence genes (toxigenic markers: *ctxA*, *ctxB*, *tcpA*, *zot*, *chxA*, *stn*; conserved virulence markers: *rtxA*, *hlyA*; adherence marker: *mshA*) were constructed and visualized as a heatmap.

Functional pathway annotation was performed using the KEGG Automatic Annotation Server (KAAS) with the bidirectional best-hit (BBH) method against the KEGG GENES database (release 2025). Annotated genes were assigned to KEGG orthology (KO) groups and mapped to corresponding functional pathway categories.

For comparative functional profiling, the same KEGG annotation pipeline was applied to the same five reference NOVC strains (973360, C3616, E1, Vc473, GXFL1-4) used in the virulence profiling analysis. The relative proportion of KEGG-annotated genes assigned to each pathway category was calculated for each strain. Global Level 1 functional architectures were compared via stacked bar charts. For Level 2 resolution, core pathways related to bacterial metabolism, environmental adaptation, and pathogenicity were selected and visualized as a row-standardized heatmap to show relative enrichment or depletion across strains.

### Average nucleotide identity analysis

Average nucleotide identity (ANI) analysis was performed to assess genomic relatedness and confirm the taxonomic affiliation of HL2601 with representative *Vibrio* strains, using the alignment-free software fastANI v1.34. The reference genomes included *V. cholerae* N16961 (GCF_900205735.1), *V. cholerae* ATCC 14035 (GCA_000621645.1), *V. vulnificus* ATCC 27562 (GCA_002224265.1), *V. alginolyticus* ATCC 17749 (GCA_000354175.2), and *V. harveyi* CAIM 1792 (GCA_000259935.1). Pairwise ANI values were calculated based on whole-genome k-mer matching. The –minFraction parameter was set to 0.05 to ensure reliable estimation for cross-species comparisons with low shared genomic fractions. A full pairwise ANI matrix was generated with the –matrix flag, and visualized as a hierarchically clustered heatmap using the pheatmap package in R v4.4.1 (Jain et al., 2018). The widely accepted threshold of 95% ANI was applied as the criterion for prokaryotic species delineation.

### Multilocus sequence typing and clonal network analysis

Multilocus sequence typing (MLST) was performed based on the standard seven-housekeeping-gene scheme for *V. cholerae* using the PubMLST database^3^ (Jolley et al., 2018). Allelic profiles of seven housekeeping genes (adk, gyrB, mdh, metE, pntA, purM, and pyrC) were determined. Novel allelic combinations were submitted to the PubMLST database curator for assignment of new sequence types.

GoeBURST analysis was conducted using PHYLOViZ 2.0 (Nascimento et al., 2017) based on the generated allelic profiles, to construct a clonal network and infer clonal relationships among sequence types.

### Phylogenetic and comparative genomic analysis

For comparative phylogenetic and genomic analysis, 37 publicly available *V. cholerae* genomes were selected following explicit inclusion and exclusion criteria to ensure analytical reliability and ecological representativeness. Inclusion criteria covered non-O1/non-O139 strains from China, South Asia and Southeast Asia with clear clinical, aquatic or foodborne isolation sources, assembly completeness >98 %, and representative strains of previously reported novel Chinese sequence types; the O1 serogroup strain N16961 was included as the outgroup. Genomes with contig N50< 10 kb (high risk of assembly artifacts), incomplete annotation, or missing serogroup or source information were excluded. After stepwise screening, all eligible genomes proceeded to core genome SNP calling and phylogenetic analysis. Detailed information on all included isolates and their assembly metrics is provided in Supplementary Table 2.

Core genome single nucleotide polymorphism (SNP) calling, filtering and core genome alignment were performed using the CSI Phylogeny v1.4 webserver^4^ (Kaas et al., 2014), with the complete genome of *V. cholerae* O1 biovar El Tor strain N16961 (GenBank accession: GCA_900205735.1) as the reference for read mapping and variant identification. Uniform filtering thresholds were applied across all isolates: minimum sequencing depth of 10×, minimum SNP quality score of 30, minimum read mapping quality of 25, minimum inter-SNP distance of 10 bp, and minimum allele frequency Z-score of 1.96. Putative recombinant regions, insertion sequences and prophages were automatically masked to avoid confounding vertical evolutionary inference. A maximum-likelihood phylogenetic tree was built using FastTree v2.1.11 under the GTR model, with node support estimated via 1000 bootstrap replicates (Price et al., 2010). The resulting tree was visualized, annotated and graphically formatted using iTOL v6.9 (Letunic and Bork, 2019).

Whole-genome synteny and structural variation were analyzed using Mauve v2.4.0 with the progressiveMauve algorithm (Darling et al., 2010). Three reference strains were selected for pairwise alignment with HL2601: the Chinese clinical NOVC isolate NOVC05, the Chinese foodborne NOVC isolate GXFL1_4, and the pandemic O1 reference strain N16961 as the outgroup. Assembly quality metrics including contig count, N50, genome completeness and contamination were calculated for all strains to assess potential assembly artifacts. A contig-aware correction algorithm was applied to exclude false inversions caused by draft assembly boundaries. Quantitative metrics including collinear block count, synteny conservation rate, inversion number/length, rearrangement breakpoints and non-collinear proportion were extracted from each alignment.

## Results

### Bacterial isolation and identification

The clinical isolate HL2601 was recovered from a patient with biliary tract infection-associated bacteremia and identified as *V. cholerae* via MALDI-TOF MS. Serogrouping assay confirmed that the isolate was negative for both O1 and O139 antigens, belonging to a non-O1/non-O139 serogroup. Gram staining presented Gram-negative, curved rod-shaped morphology consistent with *Vibrio* species.

### Phenotypic antimicrobial susceptibility

Phenotypic susceptibility testing demonstrated that HL2601 was susceptible to all 11 routinely tested antibiotics, including imipenem (susceptible, zone diameter 24 mm), cefepime (MIC ≤ 1 μg/mL), ceftazidime (MIC ≤ 1 μg/mL), ciprofloxacin (MIC ≤ 0.25 μg/mL), levofloxacin (MIC ≤ 0.25 μg/mL), gentamicin (MIC ≤ 1 μg/mL), amikacin (MIC ≤ 2 μg/mL), piperacillin-tazobactam (MIC ≤ 4 μg/mL), ampicillin (MIC ≤ 2 μg/mL), ampicillin-sulbactam (MIC ≤ 2 μg/mL), and trimethoprim-sulfamethoxazole (MIC ≤ 20 μg/mL). Quality control strains yielded MIC values within the expected ranges, validating the test results.

### Whole-genome sequencing and genomic features

*De novo* assembly of strain HL2601 yielded a high-quality draft genome consisting of 50 contigs, with a total length of 4,296,868 bp and an overall G+C content of 47.23%. The largest contig was 547,470 bp, and the contig N50 reached 324,224 bp. CheckM analysis confirmed a genome completeness of 99.98% with a contamination rate of 0.03%, indicating a contiguous, complete, and non-contaminated assembly.

The high assembly continuity (N50 > 300 kb) supports reliable detection of large-scale (>10 kb) genomic structural rearrangements. Small-scale structural variants (<10 kb) should be interpreted with caution due to inherent limitations of short-read draft assemblies.

Genomic screening for antimicrobial resistance determinants identified multiple intrinsic resistance mechanisms, including the almEFG operon (conferring polymyxin resistance via lipid A modification, an antimicrobial class not included in our susceptibility testing panel), a chromosomal *parE* homolog without known resistance-conferring mutations, and the *catB9* gene (encoding chloramphenicol acetyltransferase). The efflux pump regulator CRP was also detected. No plasmid-borne or acquired transferable resistance genes were found, consistent with the phenotypic susceptibility to all tested routine antibiotics.

### Virulence gene profiling

Virulence factor annotation against VFDB identified a total of 136 virulence-associated genes in HL2601, assigned to eight functional categories. A system-level summary of these virulence determinants, including gene counts and completeness of each functional system, is presented in Table 1; the full per-gene inventory is available in Supplementary Table 1.

**TABLE 1:** Summary of virulence-associated systems and representative genes in *V. cholerae* HL2601.

Functional category	Virulence system/determinant	Representative genes	No. of genes	Completeness	Identity range (%)	Coverage range (%)
Secretion systems	Type II secretion system (T2SS)	*epsC–N*	12	Complete	98.8–100.0	99.2–99.9
	Type VI secretion system (T6SS)	*vasA–L, hcp-2, vgrG-2*	14	Complete	93.6–100.0	99.4–99.9
	Type III secretion system 2 (T3SS2)	*vscC/N/R/S/T2, vopB2, vopD2, vcrD2*	8	Partial	90.5–96.4	98.7–99.8
Toxins	RTX toxin cluster	*rtxA–rtxD*	4	Complete	98.0–100.0	99.4–100.0
	Hemolysins/cytolysins	*tlh, hlyA (VCC)*	2	N/A	99.5–99.8	99.8–99.9
Iron uptake	Enterobactin & vibriobactin systems	*vctACD/G, irgA, viuA/D, vibA–F, hutR*	14	Complete	97.6–100.0	98.5–100.0
Adherence	MSHA type IV pilus	*mshB–mshN*	12	Complete	92.6–100.0	96.3–99.9
	Type IV pilus (partial)	*pilB, pilC, pilD*	3	Partial	98.9–100.0	99.7–99.8
Antiphagocytosis	Capsular polysaccharide	*cpsA–F, wbfU, wecC*	7	Partial	99.2–99.8	97.6–99.9
Motility & chemotaxis	Flagellar assembly & chemotaxis	*- (56 genes)*	56	Complete	94.0–100.0	96.3–100.0
Enzymes	Extracellular enzymes	*hap/vvp, nanH*	2	N/A	98.3–99.7	99.8–99.9
Quorum sensing	Autoinducer synthases	*luxS, cqsA*	2	N/A	99.7–100.0	99.4–99.7

Virulence factors were annotated against the Virulence Factor Database (VFDB) with screening thresholds of ≥90% nucleotide identity and ≥80% query coverage; identity and coverage ranges span all genes within each system. Complete, all core structural and functional genes of the system were detected; Partial, only a subset of core genes was identified. N/A (not applicable), completeness assessment is not applicable for categories consisting of individually assessed single genes, rather than defined multi-gene systems or loci. All detected virulence genes are chromosomally encoded. The full per-gene list (gene name, functional description, identity, coverage and genomic location) is provided in Supplementary Table 1. Targeted BLASTn searches (≥70% identity, ≥50% coverage) confirmed the absence of key toxigenic factors in HL2601: *ctxAB*, *tcp*, *zot*, *chxA*, and *stn*.

The strain harbored complete type II secretion system (T2SS) and type VI secretion system (T6SS) loci, as well as a partial type III secretion system 2 (T3SS2) gene cluster. Toxin-related determinants included the intact RTX toxin gene cluster (rtxA–rtxD), thermolabile hemolysin (tlh), and *V. cholerae* cytolysin (hlyA). Multiple iron acquisition pathways, represented by enterobactin and vibriobactin transport systems, were fully encoded. Additional virulence components comprised MSHA type IV pilus, a partial type IV pilus locus, capsular polysaccharide biosynthesis genes, 56 chemotaxis and flagellar genes, extracellular enzymes, and quorum sensing systems.

Targeted BLASTn searches confirmed the absence of all major toxigenic loci in HL2601, including cholera toxin (*ctxAB*), toxin-coregulated pilus (*tcp*), zonula occludens toxin (*zot*), cholix toxin (*chxA*), and thermostable enterotoxin (stn), consistent with its non-toxigenic NOVC phenotype.

To contextualize the virulence repertoire of HL2601, we compared presence/absence profiles of nine key virulence genes across five additional representative NOVC strains from clinical, environmental and aquaculture origins (Figure 1). All examined strains uniformly lacked the six classical toxigenic factors (ctxA, ctxB, tcpA, zot, chxA, stn) and consistently carried rtxA and hlyA, defining a conserved core virulence backbone of non-toxigenic *V. cholerae*. In contrast, the MSHA pilus gene mshA was detected in the majority of the included clinical isolates and the environmental isolate, but absent in HL2601 and the aquaculture water strain Vc473, indicating intraspecies variation in adherence-associated determinants among the examined NOVC strains.

**FIGURE 1:**
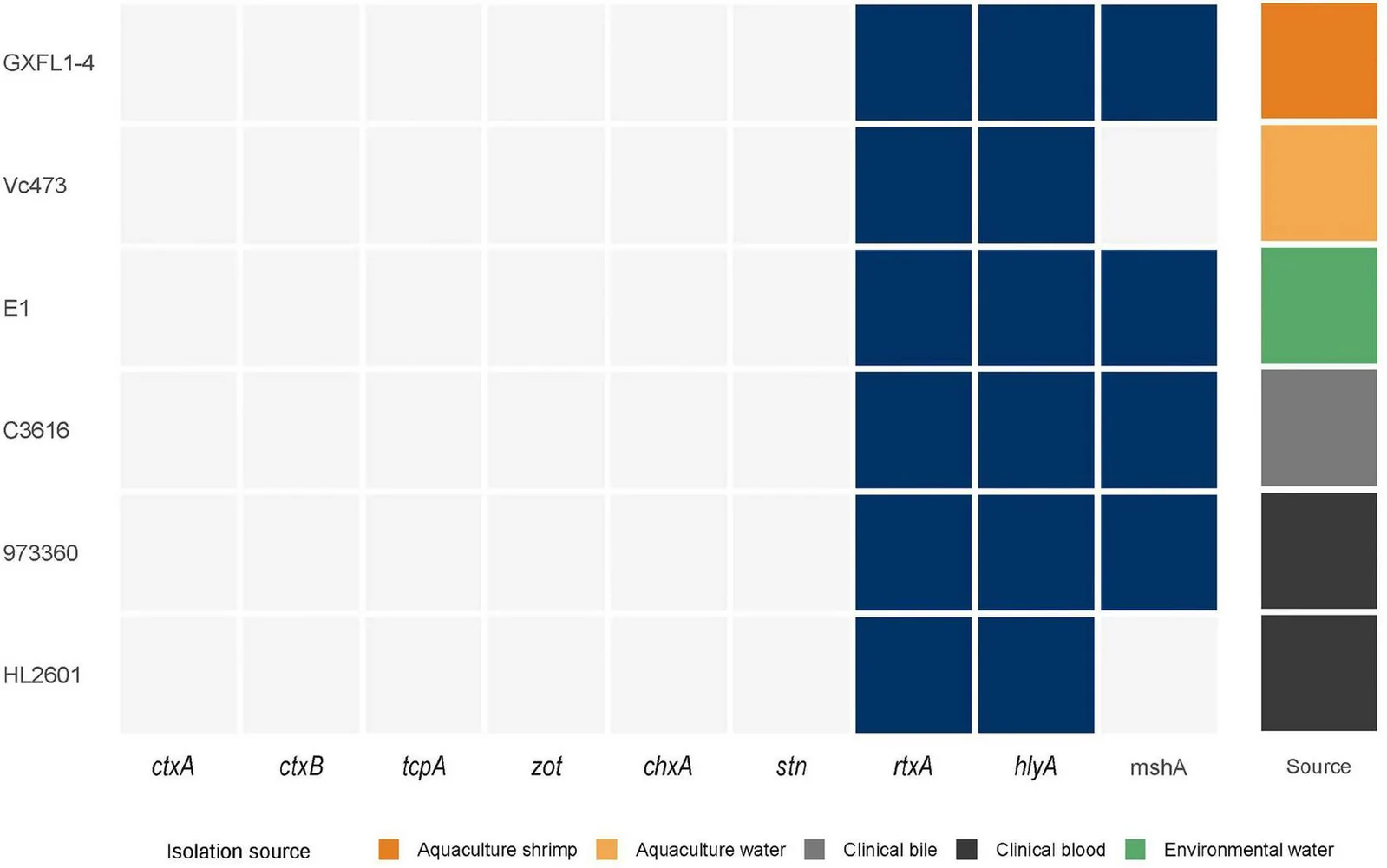
Comparative profiling of key virulence determinants across *V. cholerae* HL2601 and five representative non-O1/non-O139 strains. Dark blue squares indicate gene presence; light gray squares indicate absence. Strains are color-coded by isolation source as annotated in the legend.

### KEGG functional profiling and comparative analysis

KEGG orthology annotation identified 2,247 genes with pathway assignments in the HL2601 genome. At the Level 1 functional category level, metabolism was the most represented class (526 genes, 23.4% of annotated genes), followed by genetic information processing (397 genes, 17.7%), cellular processes (278 genes, 12.4%), environmental information processing (126 genes, 5.6%), human diseases (107 genes, 4.8%), and organismal systems (34 genes, 1.5%). This hierarchical distribution of functional categories is consistent with the general genomic organization of most free-living bacterial species.

Comparative analysis across six NOVC strains of diverse origins revealed highly conserved global functional architectures at the Level 1 category level, with no large-scale gain or loss of major functional classes observed across strains (Figure 2A). All strains consistently showed metabolism as the dominant functional category, indicating that the overall functional repertoire of HL2601 is typical of NOVC *V. cholerae*.

**FIGURE 2:**
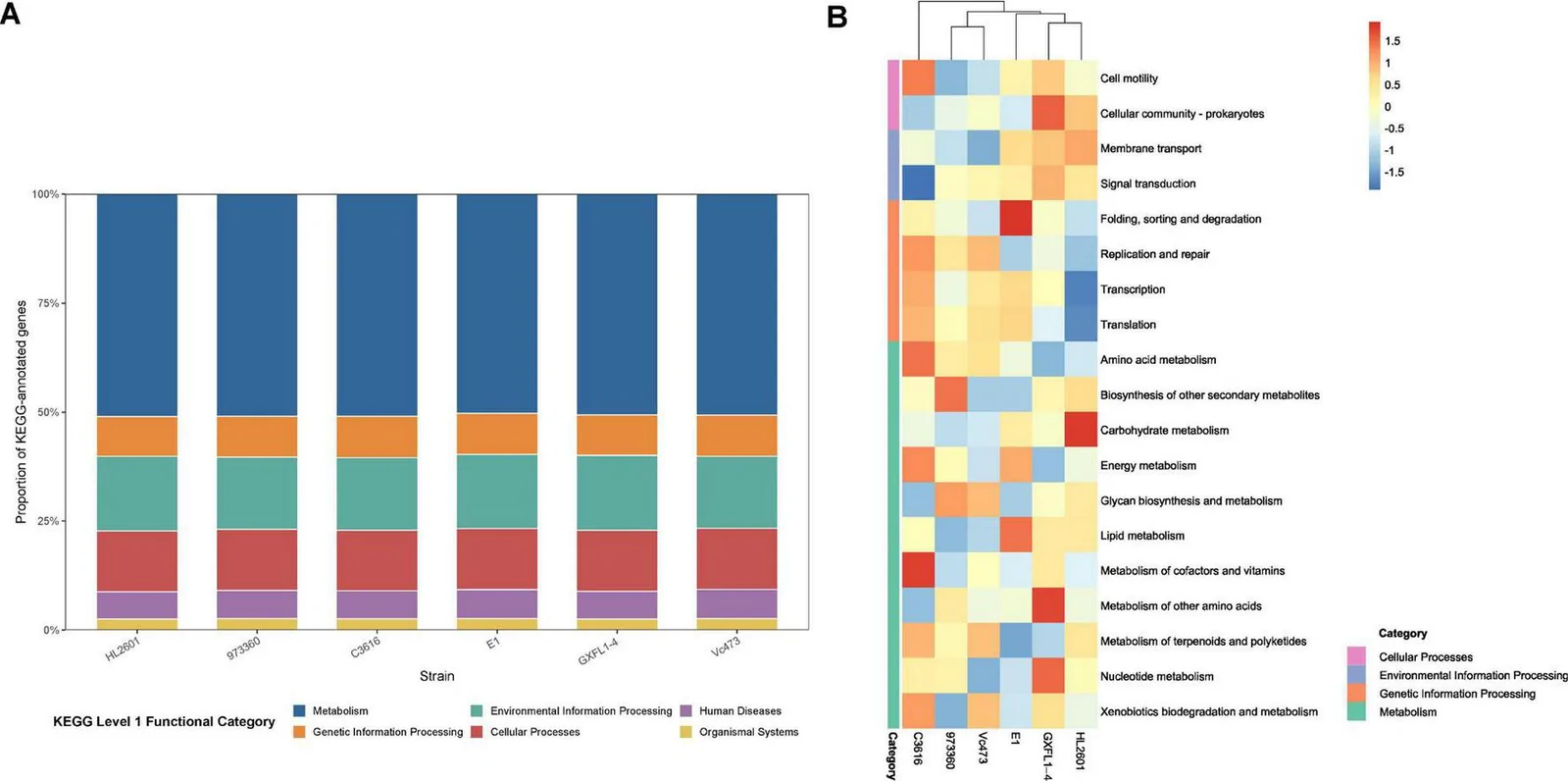
Comparative KEGG functional profiling of *V. cholerae* HL2601 and five reference non-O1/non-O139 strains. **(A)** Stacked bar chart showing the relative proportion of KEGG-annotated genes assigned to six Level 1 functional categories. All strains exhibit highly conserved global functional architecture, with metabolism as the most represented category. **(B)** Heatmap of core Level 2 pathway relative abundance. Values are row-standardized to show relative enrichment (red) or depletion (blue) of each pathway across the strain panel. Rows are grouped by Level 1 functional categories as indicated by the left-side color bar. Columns are hierarchically clustered based on overall pathway profile similarity.

At the finer Level 2 pathway resolution, subtle but consistent differences in relative pathway abundance were detectable among strains. Compared with environmental and aquaculture isolates, HL2601 showed mild relative enrichment in pathways including carbohydrate metabolism, membrane transport, and biosynthesis of secondary metabolites (Figure 2B). In contrast, pathways involved in nucleotide metabolism and replication and repair were slightly underrepresented relative to the population mean. These were quantitative differences in relative gene proportion rather than qualitative presence/absence differences, and all core functional pathways were intact in all examined strains.

### ANI analysis

Average nucleotide identity (ANI) analysis was performed against representative *Vibrio* species to confirm the taxonomic affiliation of HL2601. As shown in the pairwise ANI heatmap (Figure 3), HL2601 clustered closely with the pandemic reference strain *V. cholerae* N16961 and *V. cholerae* ATCC 14035, with ANI values of 98.11% and 98.04%, respectively. Both values are well above the 95% threshold for prokaryotic species delineation, confirming HL2601 as *V. cholerae*. In contrast, ANI values between HL2601 and other *Vibrio* species were much lower: 79.55% for *V. vulnificus*, 79.03% for *V. alginolyticus*, and 78.50% for *V. harveyi*, all well below the species cutoff and clearly differentiating HL2601 from closely related vibrios.

**FIGURE 3:**
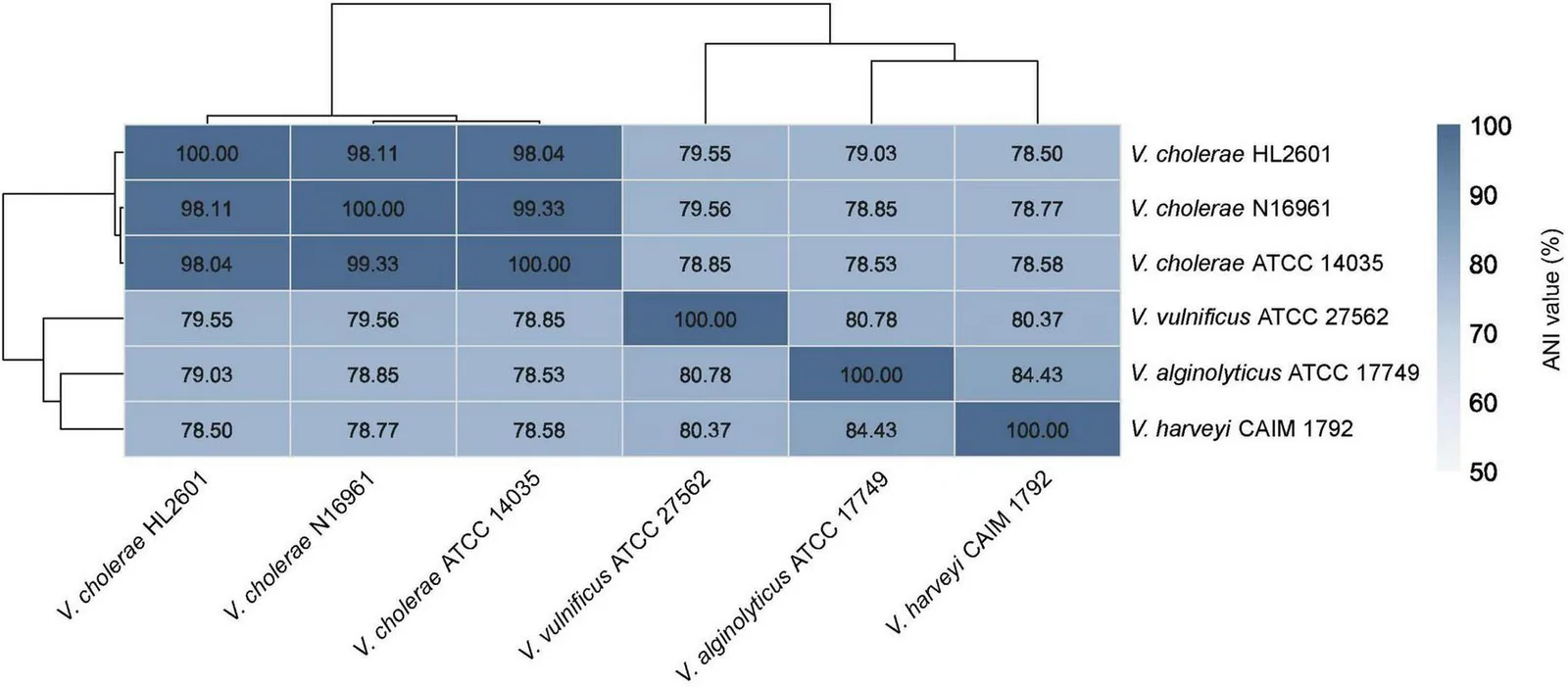
Average nucleotide identity (ANI) analysis of *V. cholerae* HL2601 and representative *Vibrio* strains. Pairwise ANI values were calculated by fastANI v1.34 and visualized as a hierarchically clustered heatmap. Color scale ranges from 50% to 100%, with 95% as the species delineation threshold.

#### Multilocus sequence typing and clonal network analysis

Multilocus sequence typing (MLST) of the seven housekeeping genes (*adk*, *gyrB*, *mdh*, *metE*, *pntA*, *purM*, and *pyrC*) yielded the allelic profile 51-41-4-79-72-1-83. This allelic combination did not match any existing sequence type in the PubMLST database and was officially assigned as a novel sequence type, designated ST2040. GoeBURST analysis based on the allelic profiles showed that ST2040 formed an independent node that was not assigned to any pre-existing clonal complex. The node was directly connected to three neighboring STs–ST1878, ST2024, and ST2042–via single-locus or double-locus variations (Figure 4).

**FIGURE 4:**
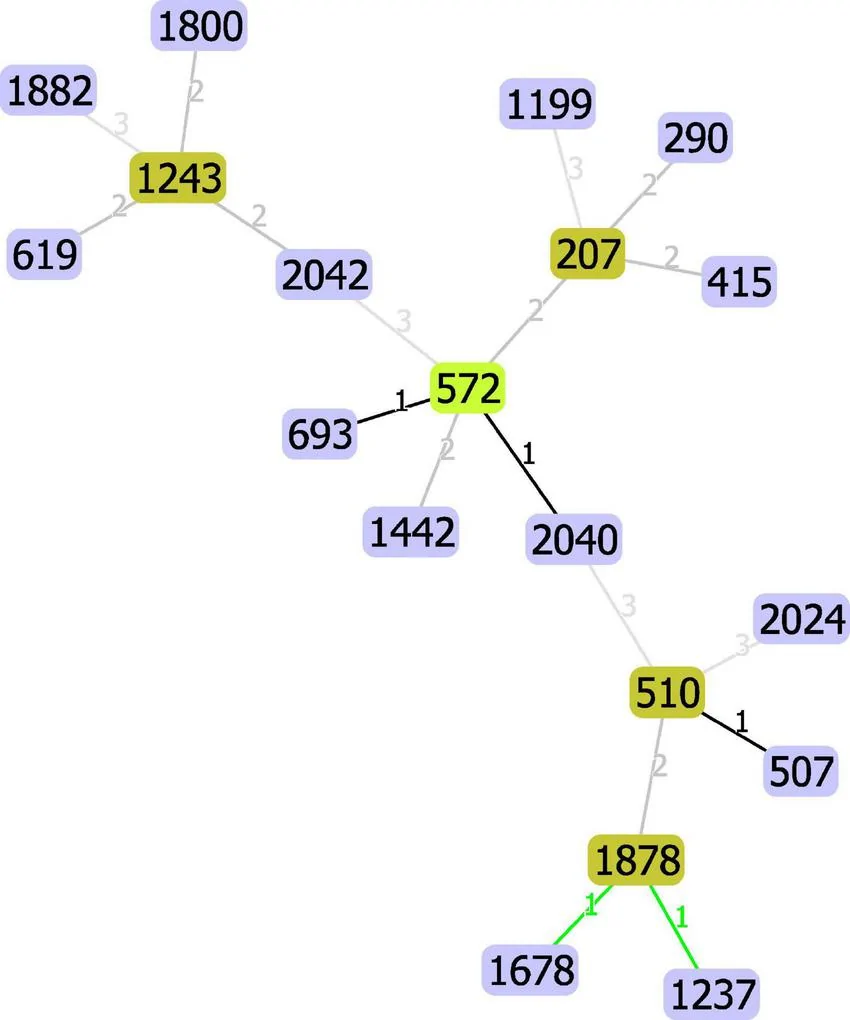
GoeBURST network analysis of ST2040 and related *V. cholerae* sequence types. The network was constructed using PHYLOViZ 2.0 based on seven-housekeeping-gene MLST allelic profiles. Nodes represent distinct sequence types; lines indicate single-locus variant (SLV) and double-locus variant (DLV) relationships.

#### Phylogenetic and comparative genomic analysis

After quality filtering and masking of recombinant and repetitive regions, a total of 3,116,207 core genome positions were retained across all 38 isolates, corresponding to 77.3% of the N16961 reference genome. The core SNP alignment was used for both maximum-likelihood phylogenetic reconstruction (Figure 5A) and principal coordinate analysis (PCoA) based on pairwise genetic distances (Figure 5B).

**FIGURE 5:**
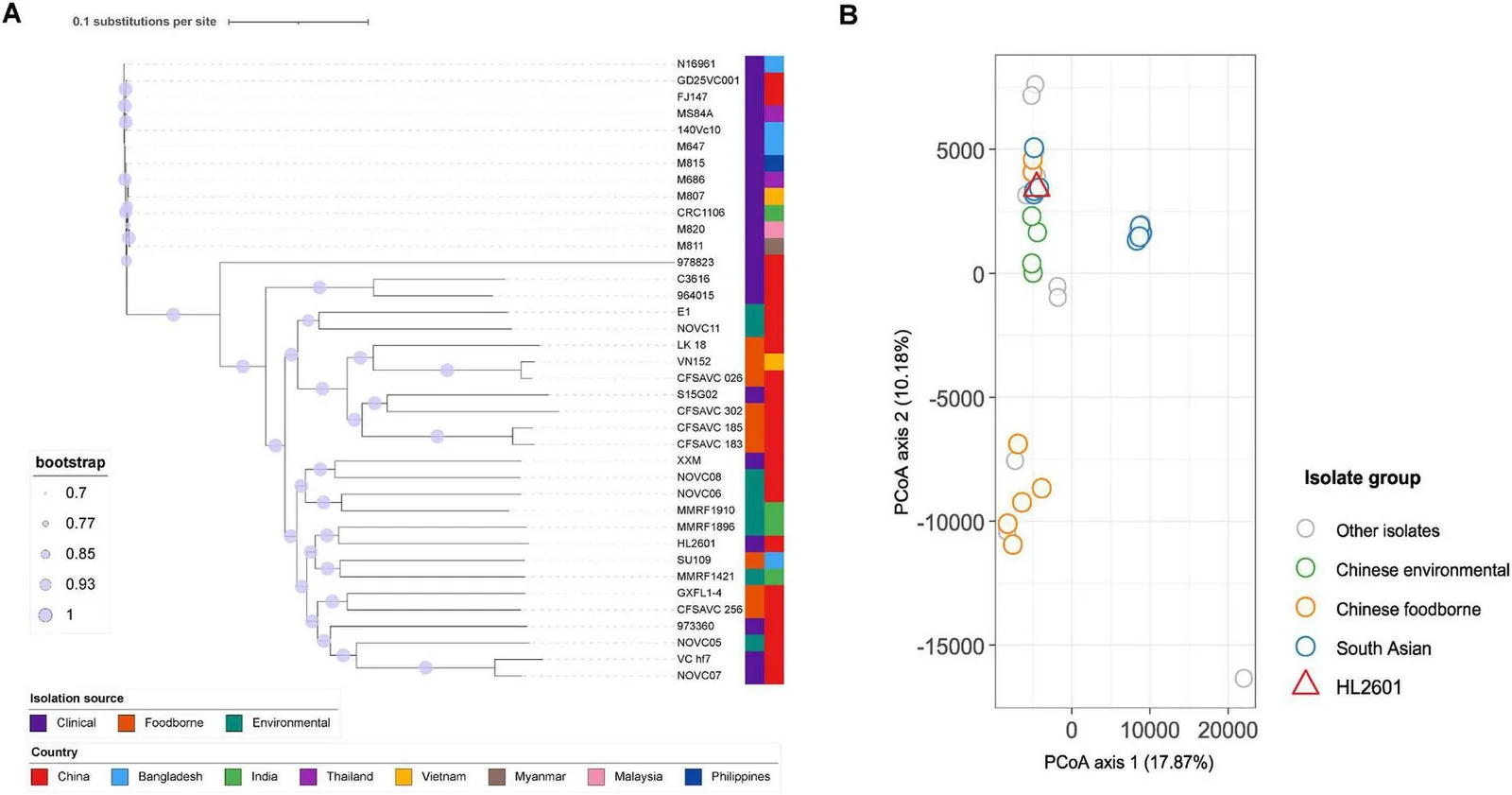
Core-genome phylogenetic reconstruction and population structure analysis of *Vibrio cholerae* HL2601 and reference isolates. **(A)** Maximum-likelihood phylogenetic tree based on core-genome SNPs. Node sizes correspond to bootstrap support values (1,000 replicates). Isolates are annotated by isolation source and geographic origin; strain N16961 is included as the outgroup. Scale bar, 0.1 nucleotide substitutions per site. **(B)** Principal coordinate analysis (PCoA) based on pairwise core-genome SNP distances. The novel isolate HL2601 is marked with a red triangle; isolates are colored by group. Axes 1 and 2 explain 17.87% and 10.18% of total genetic variance, respectively.

Phylogenetic analysis confirmed that HL2601 fell within a well-supported non-pandemic NOVC lineage, clearly separated from the O1 pandemic reference strain (Figure 5A). Within this lineage, isolates did not form strict monophyletic clades corresponding to their isolation source; instead, clinical, environmental and foodborne isolates were phylogenetically intermingled. HL2601 clustered within a broader sublineage containing extraintestinal clinical isolates (e.g., NOVC05, NOVC07, 973360), Indian environmental isolates (e.g., MMRF1896, MMRF1910) and foodborne isolates (e.g., GXFL1-4), and did not form a highly supported exclusive cluster with isolates from any single source.

Quantitative pairwise core genome SNP distances were calculated to contextualize the observed short branch lengths. The distance between HL2601 and its closest relative (Bangladesh clinical isolate M647) was 23,720 SNPs (∼0.76% nucleotide divergence across the core genome). Distances between HL2601 and the Indian environmental isolate MMRF1896, and the Chinese clinical isolate NOVC05, were 26,382 SNPs (∼0.85% divergence) and 26,309 SNPs (∼0.84% divergence), respectively.

Principal coordinate analysis was fully concordant with the phylogenetic topology, revealing three major genetic groups: (i) a divergent basal group of older Chinese clinical isolates; (ii) a major, genetically homogeneous cluster comprising most clinical, environmental and foodborne NOVC isolates from China and South/Southeast Asia, within which HL2601 was situated; and (iii) the distinct O1 pandemic outgroup. Foodborne isolates formed a tight subcluster nested within the main lineage, closely adjacent to clinical and environmental strains.

To further characterize genome structural similarity across these phylogenetic lineages, we performed whole-genome collinearity analysis (Figure 6), and quantitative synteny and structural variation statistics are summarized in Table 2. All strains exhibited high assembly quality; HL2601 had 99.98% estimated completeness and 0.03% contamination, supporting reliable detection of large-scale rearrangements. HL2601 showed the highest synteny conservation with clinical isolate NOVC05 (89.79%), with 24 genuine inversions (7,534 bp total) and 10.21% non-collinear sequence. The foodborne isolate GXFL1-4 showed 85.11% synteny conservation, comparable to the 85.52% observed with O1 pandemic strain N16961. No large-scale genuine inversions were detected between HL2601 and GXFL1-4 after contig boundary correction, whereas 20 large inversions (273,314 bp total) were identified between HL2601 and N16961. Based on full genome annotation, unaligned regions were inferred to consist of mobile genetic elements and strain-specific accessory sequences, with no acquired resistance genes or major pathogenicity islands detected. The overall gradient of structural similarity was concordant with the core genome phylogeny at the serogroup level.

**FIGURE 6:**
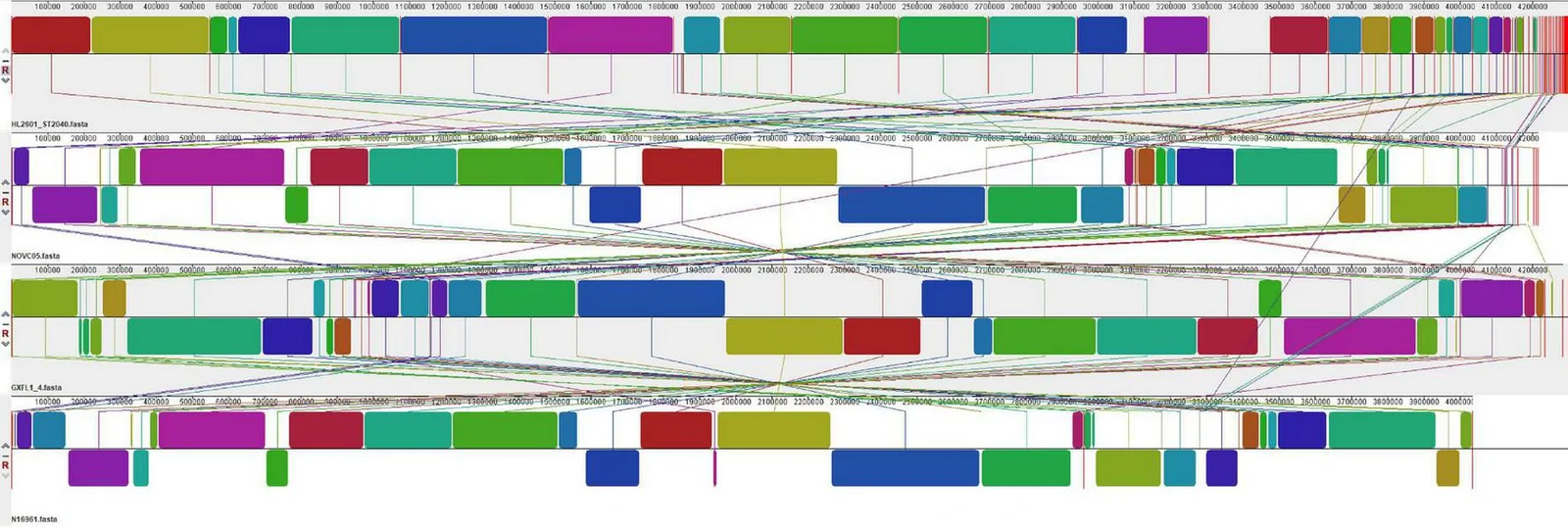
Genome collinearity comparison of *V. cholerae* HL2601 with representative reference strains. Locally collinear blocks (LCBs) are represented by colored segments, and inverted regions are shown as blocks shifted below the central line. The strains are ordered from top to bottom by decreasing phylogenetic relatedness to HL2601. Vertical red lines indicate contig boundaries of the draft assemblies.

**TABLE 2:** Quantitative statistics of genome collinearity and structural variation between HL2601 and reference strains.

Reference strain	Number of collinear blocks	Synteny conservation (%)	Number of genuine inversions	Total inverted length (bp)	Number of rearrangement breakpoints	Non-collinear length (bp)	Non-collinear proportion (%)
N16961	604	85.52	20	273,314	603	622,253	14.48
NOVC05	521	89.79	24	7,534	520	438,535	10.21
GXFL1-4	225	85.11	0	0	224	640,005	14.89

## Discussion

This study reports the genomic and phenotypic characterization of HL2601, a novel ST2040 NOVC isolate recovered from a bloodstream infection in Xiamen, southern China. The findings expand the documented genetic diversity of clinical NOVC isolates in China and shed light on the population structure and pathogenic features of emerging NOVC clones in coastal settings.

The designation of ST2040 as a novel sequence type provides further evidence for the ongoing diversification of NOVC populations in China. The very short branch lengths in the core-genome phylogeny, indicating minimal nucleotide divergence between HL2601 and its closest relatives, show that this new ST emerged from recent diversification with little overall genomic change. Quantitative pairwise core genome SNP distances further supported this pattern: HL2601 differed from its closest relatives by 23,720–26,382 core SNPs, corresponding to approximately 0.76%–0.85% nucleotide divergence across the core genome. This pattern aligns with previous observations that MLST allelic variation can accumulate rapidly to generate new STs without broad genomic divergence. Supporting this, goeBURST analysis positioned ST2040 as a peripheral node connected to neighboring sequence types via both single-locus variant (SLV) and double-locus variant (DLV) relationships, consistent with its emergence from a locally circulating clone (Nascimento et al., 2017). Of note, this study did not formally exclude the contribution of homologous recombination to the observed sequence differences, and the precise evolutionary mechanism underlying the emergence of ST2040 requires further investigation.

Phylogenetically, ST2040 clustered within a non-pandemic lineage comprising extraintestinal clinical, aquatic environmental, and foodborne isolates. This phylogenetic placement is consistent with the hypothesis of an environmental reservoir origin for this clinical strain, rather than representing a distinct, independently evolved lineage adapted to humans. This interpretation aligns with existing genomic epidemiological evidence that non-toxigenic *V. cholerae* lineages circulate independently in aquatic environments and cause sporadic human infections (Vezzulli et al., 2020). The close relatedness between clinical and environmental isolates within the same clade reinforces the view that environmental reservoirs act as a persistent source of opportunistic disease (Zhang et al., 2024). Similar findings have been reported for NOVC bloodstream isolates from other parts of China, where clinical strains showed close phylogenetic affinity to environmental isolates (Wang et al., 2024; Xu et al., 2023), suggesting this pattern is widespread across the country.

We also observed that clinical, environmental, and foodborne isolates were not separated into distinct monophyletic clades according to their isolation source; instead, they were phylogenetically intermingled. This indicates that there is no strict evolutionary boundary between environmental and clinical populations of non-pandemic *V. cholerae*, and that diverse environmental clones may have pathogenic potential under conditions of host susceptibility. The recent description of multiple novel STs from clinical NOVC isolates in China–including ST1553 (Xu et al., 2023), ST1538 (Wang et al., 2024) and the fatal ST1665 (Wang et al., 2025) –further highlights the ongoing diversification and clinical relevance of these emerging clones.

Whole-genome collinearity analysis further corroborated these phylogenetic relationships at the genomic structural level. The gradient of synteny conservation matched the core-genome phylogeny: highest between HL2601 and the closely related clinical NOVC isolate NOVC05, lower between the clinical isolate and the foodborne NOVC strain GXFL1-4, and lowest between NOVC and the distantly related O1 pandemic strain N16961 (Thompson et al., 2004; Volodarskiy and Rayko, 2026). In line with inferences from full genome annotation, non-collinear regions between serogroups comprised both large-scale chromosomal inversions and accessory genomic elements, while within the NOVC lineage unaligned segments were dominated by mobile genetic elements and strain-specific accessory sequences with no major core genome rearrangements (Heidelberg et al., 2000; Ochman et al., 2000). This pattern reflects differing evolutionary depths: between O1 and NOVC, extensive structural divergence points to deep, long-term independent evolution of the two lineages (Heidelberg et al., 2000). Within the NOVC clade, by contrast, the conserved core genome backbone and accessory-driven structural variation are consistent with relatively recent diversification of the lineage (Chun et al., 2009). No acquired antimicrobial resistance genes or major pathogenicity islands were identified in the non-collinear regions, indicating that horizontal gene transfer of key resistance or virulence determinants is not a major driver of structural differentiation among these strains (Ochman et al., 2000).

The virulence gene profile of ST2040 demonstrates pathogenic potential beyond the simple absence of cholera toxin. The presence of an intact type II secretion system (T2SS) and type VI secretion system (T6SS), together with RTX toxin, hemolysins and multiple iron uptake systems, indicates that this non-toxigenic strain relies on alternative virulence mechanisms for extraintestinal pathogenesis (Crisan and Hammer, 2020; Lin et al., 1999). The T6SS is of particular note, as it mediates interbacterial competition, immune evasion and host cell cytotoxicity–functions that support colonization and survival in extraintestinal niches (Crisan and Hammer, 2020). A partial T3SS2 locus was also identified; although incomplete relative to the functional system in *V. parahaemolyticus*, its presence adds to the strain’s accessory virulence repertoire, even if its functional contribution remains unconfirmed (Dziejman et al., 2005). Hemolysins (TLH and VCC) and RTX toxin may contribute to tissue damage and survival in the bloodstream, while iron uptake systems enable nutrient acquisition in the iron-limited host environment (Lin et al., 1999; Ruenchit et al., 2017). Together, these factors provide a plausible mechanistic basis for the ability of non-toxigenic NOVC to cause severe invasive infection, especially in immunocompromised individuals (Zhang et al., 2024).

KEGG pathway analysis provided functional context for the strain’s metabolic capacity, and comparative profiling across five reference NOVC strains (clinical, environmental and foodborne origins) was performed to identify relative functional differences (Figure 2). At the Level 1 functional category level, the proportional distribution of annotated genes was highly conserved across all six examined strains, with metabolism as the largest category, followed by genetic information processing and cellular processes. This global functional architecture is typical of most free-living bacterial species and is not specific to HL2601. At the finer Level 2 pathway resolution, HL2601 showed mild relative enrichment in carbohydrate metabolism, membrane transport, and biosynthesis of secondary metabolites compared with the reference strain panel, while pathways involved in nucleotide metabolism and replication and repair were slightly underrepresented. These were quantitative differences in relative gene abundance rather than qualitative presence or absence of entire functional pathways.

The relative enrichment of carbohydrate metabolism pathways suggests that the strain maintains robust capacity for utilizing diverse carbon sources, which may confer an advantage in niche adaptation in aquatic environments and host mucosal surfaces. Genes annotated to human disease pathways were detected at low proportions and were universally present across all examined strains; this annotation alone does not confirm pathogenic function and requires further experimental validation.

The patient’s clinical profile–poorly controlled diabetes, chronic heavy alcohol use and structural biliary tract abnormalities–represents well-recognized risk factors for NOVC bacteremia in China (Li et al., 2020). Biliary tract disease most likely facilitated retrograde entry of bacteria from the gut into the bloodstream, leading to secondary sepsis in the setting of hepatic dysfunction and impaired immunity associated with chronic alcohol consumption. The absence of cholera-like diarrhea in this patient confirms the extraintestinal disease pattern typical of NOVC, distinct from toxigenic O1/O139 strains, and underscores the need to consider NOVC in the differential diagnosis of bacteremia in high-risk patients without gastrointestinal symptoms.

HL2601 showed phenotypic susceptibility to all routinely tested antibiotics, consistent with the typical profile of environmental *V. cholerae* isolates that lack acquired resistance determinants (Kitaoka et al., 2011). We also identified intrinsic efflux pump regulators and a chromosomal *parE* homolog lacking known resistance-conferring mutations. Although these determinants do not confer clinical resistance, they may serve as a genetic reservoir for the evolution of higher-level fluoroquinolone resistance under antimicrobial selective pressure (Kitaoka et al., 2011). Efflux pumps in Vibrionaceae contribute not only to antimicrobial tolerance but also to virulence, persistence and biofilm formation, making them relevant to both treatment outcomes and pathogenic potential (Kumar et al., 2023). For empirical treatment in this region, conventional β-lactams (including extended-spectrum cephalosporins) and fluoroquinolones remain appropriate first-line options.

This study has several limitations. Regarding sample representativeness and source tracing, it is based on a single clinical isolate, which cannot capture the full population structure or prevalence of this novel ST in the region and limits generalizability; detailed source-tracing data, including environmental sampling from the patient’s residential area and suspected food sources, are unavailable, so the inferred environmental reservoir origin is based primarily on phylogenetic clustering, and although the reference strain panel covers clinical, environmental, and foodborne sources, it cannot fully represent the overall ecological and geographic diversity of NOVC populations. In terms of technical limitations of genomic analysis, this study relies on short-read draft genome assemblies, such that small-scale structural variants (<10 kb) may be affected by assembly fragmentation while large-scale collinearity features and chromosomal rearrangement conclusions remain reliable; in addition, homologous recombination was not formally excluded from the core genome SNP analysis, which may have a minor impact on the estimation of pairwise evolutionary distances. Finally, the partial type III secretion system 2 (T3SS2) locus identified here requires further functional characterization to determine whether it contributes to virulence. Broader surveillance of NOVC isolates from both clinical and environmental sources in coastal China is warranted in future work to better define the population structure and transmission dynamics of this opportunistic pathogen.

## Conclusion

We report the first identification of the novel sequence type ST2040 of non-O1/non-O139 *V. cholerae* causing bloodstream infection in Xiamen, southern China. The isolate lacks cholera toxin but harbors multiple alternative virulence factors, including complete T2SS and T6SS, RTX toxin, hemolysins, and iron uptake systems, suggesting extraintestinal pathogenic potential. The extremely short phylogenetic branch length and small core-genome SNP distance to its closest relatives are consistent with the emergence of ST2040 likely through recent point mutations in housekeeping genes. The universal phenotypic susceptibility to clinically relevant antibiotics provides reassurance for empirical therapy, while the presence of intrinsic resistance determinants highlights the value of both genomic and phenotypic characterization.

Whole-genome collinearity analysis further demonstrated high structural conservation between HL2601 and clinical NOVC isolates, with structural divergence from foodborne isolates driven primarily by accessory genome gain and loss rather than large chromosomal rearrangements.

Integration of whole-genome sequencing and MLST into routine *V. cholerae* surveillance in coastal regions, combined with targeted health education for high-risk populations, will be essential for timely detection of emerging NOVC clones and reduction of the burden of extraintestinal NOVC infections.

## Data Availability

The datasets presented in this study can be found in online repositories. The names of the repository/repositories and accession number(s) can be found below: https://www.ncbi.nlm.nih.gov/, PRJNA1479141, https://www.ncbi.nlm.nih.gov/, SRR39195233.

## Footnotes

1
https://github.com/tseemann/barrnap

2
https://cge.food.dtu.dk/services/ResFinder/

3
https://pubmlst.org

4
https://cge.food.dtu.dk/services/CSIPhylogeny/

## Supplementary material

Supplementary Table 1 Full inventory of virulence-associated factors in *Vibrio cholerae* HL2601, including functional classification, gene annotation, nucleotide identity, query coverage and genomic location.

Supplementary Table 2 Detailed information of 38 *Vibrio cholerae* isolates included in the core-genome phylogenetic and comparative genomic analyses, covering isolation source, geographic origin, GenBank accession numbers and assembly quality metrics.
